# ImbDef-GAN: Defect Image-Generation Method Based on Sample Imbalance

**DOI:** 10.3390/jimaging11100367

**Published:** 2025-10-16

**Authors:** Dengbiao Jiang, Nian Tao, Kelong Zhu, Yiming Wang, Haijian Shao

**Affiliations:** 1 School of Computer Science, Jiangsu University of Science and Technology, Zhenjiang 212003, China; 231210702111@stu.just.edu.cn (N.T.); 231210701133@stu.just.edu.cn (K.Z.); jsj_shj@just.edu.cn (H.S.); 2 Tofflon Science & Technology Group Co., Ltd., Shanghai 200000, China; yiming@tofflon.com

**Keywords:** deep learning, sample imbalance, background image generation, defect image generation, defect features

## Abstract

In industrial settings, defect detection using deep learning typically requires large numbers of defective samples. However, defective products are rare on production lines, creating a scarcity of defect samples and an overabundance of samples that contain only background. We introduce ImbDef-GAN, a sample imbalance generative framework, to address three persistent limitations in defect image generation: unnatural transitions at defect background boundaries, misalignment between defects and their masks, and out-of-bounds defect placement. The framework operates in two stages: (i) background image generation and (ii) defect image generation conditioned on the generated background. In the background image-generation stage, a lightweight StyleGAN3 variant jointly generates the background image and its segmentation mask. A Progress-coupled Gated Detail Injection module uses global scheduling driven by training progress and per-pixel gating to inject high-frequency information in a controlled manner, thereby enhancing background detail while preserving training stability. In the defect image-generation stage, the design augments the background generator with a residual branch that extracts defect features. By blending defect features with a smoothing coefficient, the resulting defect boundaries transition more naturally and gradually. A mask-aware matching discriminator enforces consistency between each defect image and its mask. In addition, an Edge Structure Loss and a Region Consistency Loss strengthen morphological fidelity and spatial constraints within the valid mask region. Extensive experiments on the MVTec AD dataset demonstrate that ImbDef-GAN surpasses existing methods in both the realism and diversity of generated defects. When the generated data are used to train a downstream detector, YOLOv11 achieves a 5.4% improvement in mAP@0.5, indicating that the proposed approach effectively improves detection accuracy under sample imbalance.

## 1. Introduction

Defect detection is critical to ensuring industrial product quality and is directly linked to product reliability and safety. Automated visual defect inspection [1] provides substantial benefits by accurately detecting and localizing defects in images, thereby reducing labor costs and improving production efficiency. However, deep learning-based defect-detection methods [2,3,4], such as YOLO [5] and Faster R-CNN [6], typically require large, diverse, and well-annotated training datasets. Collecting defective samples poses two challenges. First, defective products are rare, yielding few samples and limited defect diversity. Second, defects are often complex and occupy only a small portion of the image, which complicates accurate annotation [7]. Consequently, some approaches adopt unsupervised training on defect-free images [8]. Without defect images, these models typically determine only whether a defect is present and struggle to distinguish categories, making them inadequate for defect classification. Data augmentation helps mitigate the scarcity and limited diversity of defect samples. As summarized in Table 1, data augmentation for defect images can be grouped into three categories: transformation-based augmentation, traditional defect image generation, and deep learning-based defect image generation.

Transformation-based approaches apply operations such as scaling, rotation, translation, and flipping to augment the dataset and may also paste cropped defect patches onto background images [9,10,11]. Although straightforward to implement, these methods struggle to capture the complexity of real defects, and the resulting defects often lack realism. With advances in deep learning, numerous methods for generating industrial surface defect images have emerged and can be broadly categorized into diffusion-based approaches and GAN-based approaches. Diffusion models, particularly denoising diffusion probabilistic models (DDPMs) [12], with DDPMs commonly adopted as a diffusion baseline due to stable training under a likelihood-based objective, relatively comprehensive mode coverage, and strong perceptual quality on image-generation benchmarks, comprise two processes: (i) a forward diffusion that progressively adds Gaussian noise until the signal approaches random noise; and (ii) a reverse generative process that removes noise stepwise to synthesize an image. However, the iterative denoising schedule yields low sampling efficiency. Without task-specific conditioning or edge-aware objectives, DDPMs may produce softened boundaries between defects and the background and less faithful high-frequency local textures. Generative adversarial networks (GANs) [13] and their variants [14,15,16,17] leverage adversarial learning and are widely used for image generation. StyleGAN3 delivers state-of-the-art, high resolution image generation with fine grained texture fidelity and realistic structural detail, while its alias-free architecture improves geometric consistency and reduces texture artifacts. However, the scarcity of defect samples constrains the ability of GAN models to learn faithful defect distributions in this domain.

In industrial production, background images are abundant, whereas defect images are scarce because such images occur infrequently and are costly to acquire. This leads to severe class imbalance, and defects often exhibit complex, irregular structures. Although recent approaches, including DFMGAN [18], AnomalyDiffusion [19], and related methods [20,21,22,23,24], can produce visually plausible defect images, several limitations remain: (i) generated defect patterns are often overly similar and fail to capture the diversity observed in production settings; (ii) edge details around defect regions are frequently lost, undermining realism; (iii) misalignment may occur between a generated defect and its corresponding mask, degrading the quality of the generated regions; and (iv) defects may appear in invalid areas, such as outside the product region. Consequently, generating realistic and diverse defect images under highly imbalanced conditions, in which background images far outnumber defect images, remains an open challenge.

**Figure 1:**
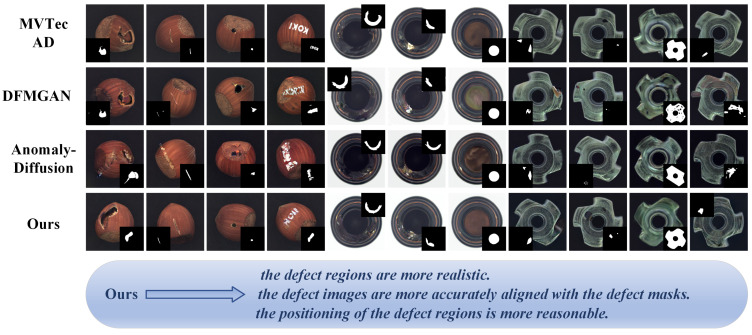
Qualitative comparison along three factors: (i) realism of defect regions; (ii) image–mask alignment; (iii) plausibility of placement.

To mitigate the aforementioned challenges in defect image generation, we propose ImbDef-GAN, a generative framework that accounts for sample imbalance. By leveraging abundant background imagery alongside scarce defect exemplars, ImbDef-GAN increases defect diversity, preserves fine-grained edge details, enforces precise correspondence between each generated image and its mask, and restricts defect placement to semantically valid regions. Representative results are shown in Figure 1. When used to train a downstream detector (YOLOv11 [25]), the generated data yield a 5.4% improvement in mAP@0.5, confirming the effectiveness of ImbDef-GAN. The proposed method consists of two components, background image generation and defect image generation, and offers the following contributions:

(1) A lightweight StyleGAN3 [17] variant jointly generates the background and a background mask, while a matching discriminator enforces coherence between the generated background and mask. The Progress-coupled Gated Detail Injection (PGDI) module regulates detail strength according to training progress, raising high-frequency fidelity while maintaining training stability. The coupling of joint mask generation with coherence supervision and progress-driven detail control yields more realistic backgrounds and enables the subsequent defect-generation stage to capture complete morphology and sharper boundaries.
(2) To mitigate unnatural transitions at defect boundaries under sample imbalance, the defect stage augments the background generator with a residual defect-feature branch, while a smoothing coefficient blends defect features with background features, yielding more natural boundaries and more realistic defect regions.
(3) To address misalignment between defect masks and generated images, a mask-aware matching discriminator propagates mask information through a multilayer feature extractor, using an explicit image–mask matching signal to enforce layer-wise alignment and strengthen spatial localization of defect regions.
(4) To increase the diversity of defect regions and avoid generation in invalid background areas, Edge Structure Loss (ESL) promotes boundary-aware morphological variation, while Region Consistency Loss (RCL) restricts the defect mask to the valid region of the background mask. Taken together, these objectives provide a unified treatment of boundary structure and region validity under sample imbalance.

The remainder of this paper is organized as follows. Section 2 reviews related work on few sample image generation and defect image generation. Section 3 describes the proposed algorithms and model architecture. Section 4 reports the experimental setup and results. Finally, the work is concluded in Section 5.

## 2. Related Work

### 2.1. Few Sample Image Generation

When training data are extremely scarce (fewer than 10 images), models readily overfit and the generated images closely resemble the training samples. A common remedy is transfer learning, which adapts a model pretrained on a large source domain to a small target domain. FreezeD [26] mitigates overfitting during fine tuning by freezing higher-level weights of the pretrained GAN discriminator and combining this with regularization and data augmentation. WeditGAN [27] learns fixed offsets in the StyleGAN latent space to construct a target latent space, preventing mode collapse and overfitting with minimal fine tuning. AdAM [28] introduces adaptation-aware kernel modulation: it identifies salient convolutional kernels via importance estimation and applies low rank modulation during fine tuning to preserve and adapt source domain knowledge, yielding stable, high quality few-shot image generation.

In contrast, few-shot customization on pretrained diffusion models has attracted wide attention. Textual Inversion [29] learns a compact set of “pseudo-token” embeddings that encode novel concepts in the text space, balancing faithfulness and editability and improving visual fidelity and editing robustness. DreamBooth [30] personalizes text to image diffusion models by fine tuning a pretrained model and introducing a class specific prior preservation loss generated by the model itself, enabling high-quality and diverse images across varied scenes, poses, and lighting conditions. Although these approaches can produce realistic images, they lack mechanisms for precise defect localization and for high quality mask generation. This limitation hinders the creation of accurate masks for downstream detection or segmentation, which is essential for anomaly image generation. Our method instead generates diverse, high-quality defect images together with their corresponding masks under few-shot conditions.

### 2.2. Defect Image Generation

The scarcity of defective samples has motivated research into defect image generation. DeVries et al. [9] simulate defects by introducing random cutouts (masked regions) into defect-free images. CutPaste [10] copies a patch from a defect-free image and pastes it at a new location to create a synthetic anomaly. Similarly, Crop&Paste [11] crops defect regions from defect images and pastes them onto other images. However, these traditional approaches produce only a limited number of samples that scale with the dataset size. They do not introduce new defect modes. Instead, they transplant existing defects onto different images.

For deep learning approaches, defect image generation can be broadly grouped into diffusion and GAN methods. Diffusion models are widely used for their strong generalization ability. Wang et al. [31] propose a progressive training diffusion model for defect image generation that improves training stability and produces diverse, high quality defect images. DualAnoDiff [32] employs two interrelated diffusion branches to jointly generate the whole image and the anomaly part, improving realism and diversity in the few-shot regime. AnomalyDiffusion [19], built on a latent diffusion model, introduces Spatial Anomaly Embedding to decouple defect appearance from location and employs Adaptive Attention Reweighting to dynamically upweight low-salience defect regions during generation. Because training focuses on masked regions, fusion between defects and the surrounding background is often inadequate, yielding poor boundary transitions and weak textural consistency. Defects may also be placed in invalid or semantically mismatched background areas, which undermines the realism of the generated images.

GANs [13] and their variants [14,15,16,17] are widely used for a wide range of image-generation tasks because of their strong generative ability. A GAN consists of a generator and a discriminator trained adversarially in a minimax game that approaches a Nash equilibrium. This pressure drives the generator to produce increasingly realistic images. The generator seeks to fool the discriminator by producing images indistinguishable from real data, while the discriminator learns to distinguish generated images from real ones.

SDGAN [20] uses two generators to transfer features between background and defect images. However, it is mainly suited to large defects that span most of the image and is less effective at generating small defects. Defect-GAN [21] follows a damage–recovery paradigm: the damage stage injects defects into defect-free images, and the recovery stage removes them to reconstruct normal images. It uses a layer-wise generation architecture that can generate realistic defects across backgrounds with diverse textures and appearances. Although Defect-GAN works when data are relatively abundant, it is not well-suited to few-shot datasets, and its outputs lack associated defect masks, which limits its applicability to detection and segmentation. Liu et al. [22] proposed Anomaly-GAN, which integrates a mask pool, an anomaly-aware loss, and both local and global discriminators, markedly improving train surface defect-detection performance. DG2GAN [23] is a framework based on generative adversarial networks that employs two generator networks together with a gradient-guided diversity module to synthesize diverse, high-fidelity defect instances while preserving background appearance. He et al. [24] proposed a StyleGAN2-ADA few-shot defect-generation method that disentangles defect-related factors to improve the realism of generated samples under limited supervision. DFMGAN [18] addresses data scarcity by pasting defect regions onto standard images to construct synthetic defective images. It first trains on standard images and then fine-tunes on defective images to enable defect image generation. While improving visual quality and easing scarcity, this strategy uses per-pixel addition to composite defects, which frequently introduces color mismatches and boundary discontinuities that degrade boundary naturalness, overall realism, and image–mask alignment. By comparison, under class imbalance, ImbDef-GAN produces defects with more natural boundaries, better aligned masks, and more plausible placement, achieving higher realism and diversity.

## 3. Methods

Generative adversarial networks (GANs) require sufficient defect samples to learn the underlying data distribution and to generate novel, high quality defect images. However, because background samples are abundant, whereas defect samples are scarce, GAN training often yields suboptimal image quality and overfits to the few available defect instances. To address this, we propose ImbDef-GAN, which improves defect image generation through a sample imbalance compensation mechanism. ImbDef-GAN mitigates these issues through a two-stage design of “background prior—defect injection”, first learning a background prior from abundant background images to obtain a stable and reusable representation, then freezing the background backbone during defect generation and injecting defect features into the generated background via a mask conditioned residual branch. This localization of trainable parameters to defect regions markedly contracts the effective hypothesis space, curbs overfitting at its source, and enables more effective use of background samples. We (1) exploit abundant background samples to compensate for limited defect data, and (2) jointly learn and fuse representations from defect and background samples to generate realistic defect images.

### 3.1. Background Image Generation

**Figure 2:**
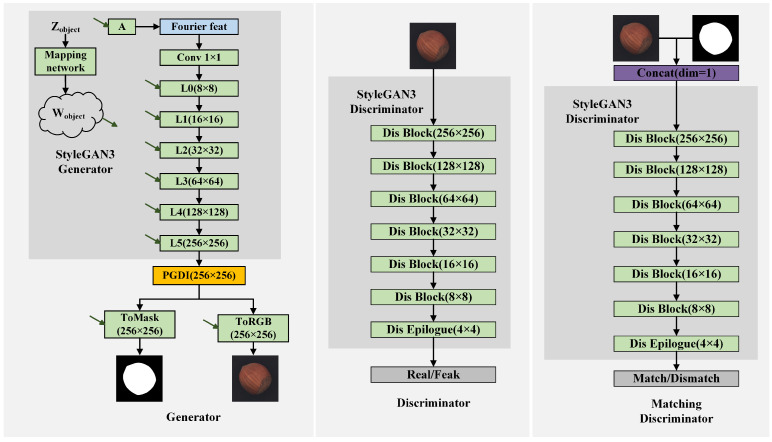
Background image generation architecture. Green arrows indicate the $W_{\mathrm{object}}$ for background features.

Compared with StyleGAN2 [16], StyleGAN3 [17] provides better control of high-frequency details and produces sharper images. However, its deeper architecture is primarily designed for high resolution face and animal generation (such as 1024 × 1024). When applied directly to background image generation, it tends to produce blurry outputs and unstable convergence, which in turn leads to loss of detail in subsequent defect masks and hinders the faithful capture of complete defect characteristics. To address these issues while preserving the underlying generative paradigm, a lightweight variant of StyleGAN3 (Figure 2) is designed. A ToMask branch is also introduced that is architecturally identical to ToRGB except for its channel dimension, enabling joint synthesis of the background image and its mask. Moreover, unregulated detail injection at early stages exacerbates high frequency noise and impairs mask learning; accordingly, the Progress-coupled Gated Detail Injection (PGDI) module adaptively modulates detail strength throughout training to balance fidelity and stability.In addition, image-level discrimination provides insufficient constraints on image to mask correspondence. A matching discriminator explicitly evaluates pairwise consistency and enforces spatial alignment.

In StyleGAN3, the spatial resolution of each generator layer is controlled by parameters such as cutoff frequency, stopband frequency, sampling rate, and target output resolution. By adjusting the layer count and these parameters, the framework configures the generator to produce background images at the desired resolution. For 256 × 256 backgrounds, the framework reduces the generator depth from 14 to 6 layers. The stages follow a power-of-two ($2^{n}\left(n\in N^{+}\right)$) growth schedule, doubling the spatial resolution at each stage. This facilitates subsequent defect generation by enabling more effective learning of defect region features.

#### 3.1.1. Progress-Coupled Gated Detail Injection Module

**Figure 3:**
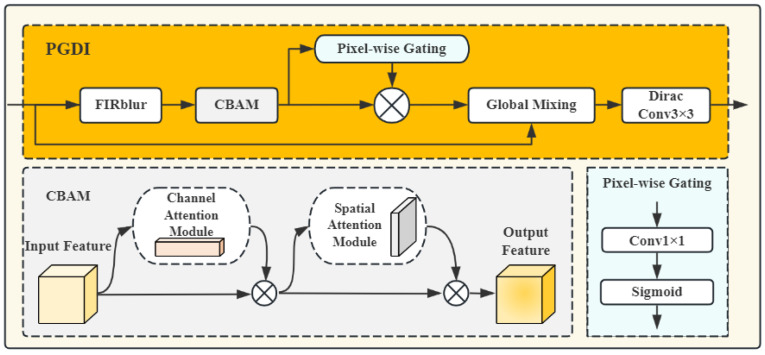
Progress-coupled Gated Detail Injection (PGDI) module.

The progress-coupled gated detail injection (PGDI) module is illustrated in Figure 3. PGDI maps training progress to a monotonic scheduling signal, driving pixel-wise gating to progressively inject high frequency components along the residual path, thereby achieving synchronized refinement of the image and mask branches and enabling dynamic control of the balance between stability and fidelity.

Let the input and output features be $x\in R^{B\times C\times H\times W}$ and $y\in R^{B\times C\times H\times W}$, respectively. To suppress noise in the input features, FIRBlur constructs a normalized separable 2D kernel from the set of 1D filter coefficients ${\left\{t_{i}\right\}}_{i=0}^{K-1}$ (Equation (1)). The input *x* is then padded by asymmetric reflection to achieve the same output size, yielding $\hat{x}$, and the kernel is applied channel-wise via depthwise separable convolution to produce the blurred features $x_{fb}\in R^{B\times C\times H\times W}$ (Equation (2)).

After denoising, $x_{fb}$ is reweighted by a Channel Attention Module (CAM) and a Spatial Attention Module (SAM) [33] to emphasize informative channels and salient spatial locations. This dual attention enables precise localization of critical feature regions and recovery of fine-grained edges, producing the enhanced features $x_{ca}\in R^{B\times C\times H\times W}$. To further regulate the injection strength of high-frequency details, we design a pixel-wise gating mechanism: a 1 × 1 convolution followed by a sigmoid activation is applied to produce a per-pixel gate map $m\in R^{B\times C\times H\times W}$. This map adaptively determines the retention of the residual augmentation branch according to local semantics. Finally, the pixel-wise gated features are fused with the original features using the global mixing coefficient to obtain the final output $x_{mix}\in R^{B\times C\times H\times W}$, as shown in Equation (4).This design enables precise high frequency injection in later training while reverting to a smoothing pathway in homogeneous regions, thereby balancing detail enhancement with training stability. For global mixing, the training progress *k* (measured in kimg) and a learnable scalar $\lambda_{raw}$ are combined via Equation (3) to compute the progress dependent mixing coefficient $\lambda^{\left(k\right)}$.

(1) $$G_{u,v}=\frac{t_{u}t_{v}}{\sum_{p=0}^{K-1}\sum_{q=0}^{K-1}t_{p}t_{q}}\;u,v=0,\ldots ,K-1$$

(2) $$x_{fb}\left[b,c,i,j\right]=\sum_{u=0}^{K-1}\sum_{v=0}^{K-1}G_{u,v}\;\hat{x}\;\left[b,c,\;i+u-\left\lfloor \frac{K}{2}\right\rfloor ,\;j+v-\left\lfloor \frac{K}{2}\right\rfloor \right]$$

(3) $$\lambda^{\left(k\right)}=\beta \lambda^{\left(k-1\right)}+\left(1-\beta \right)\left[\text{clamp}\;\left(\frac{k}{K_{prog}},0,1\right)\times \sigma \left(\lambda_{raw}\right)\right]$$

(4) $$x_{mix}=\lambda^{\left(k\right)}\left(m⊙x_{ca}\right)+\left(1-\lambda^{\left(k\right)}\right)x$$

where $\hat{x}$ denotes the input feature map after independent reflection padding on the top, bottom, left, and right. *k* is the current training progress. $K_{prog}$ controls the amount of progress required for the mixing coefficient $\lambda^{\left(k\right)}$ to “warm start” from 0 to its maximum (we set $K_{prog}=100$ kimg) and σ(·) denotes the sigmoid function. β=0.99.

Finally, the fused features $x_{mix}$ are passed through a 3 × 3 convolution layer initialized with Dirac weights to produce the output features $y\in R^{B\times C\times H\times W}$. This initialization behaves as an approximate identity mapping at early training, stabilizing feature propagation. As training proceeds, the convolution weights adaptively learn an optimal filtering response, suppressing high-frequency noise while preserving fine textures. With this design, the PGDI module injects high-frequency details into the features in a controlled manner while maintaining training stability.

#### 3.1.2. Matching Discrimination

By jointly evaluating image and mask pairs, a matching discriminator (Figure 2) explicitly couples the background and mask outputs and enhances spatial consistency, thereby enforcing alignment, improving localization accuracy, and reducing boundary artifacts. The matching discriminator takes four channels as input: the first three are the generated RGB background, and the fourth is the corresponding single-channel background mask. Its architecture mirrors that of the original discriminator, preserving compatibility with the existing adversarial training pipeline. During training, the matching discriminator distinguishes real from generated pairs of images and masks. To jointly enhance the realism of background images and the spatial alignment of masks, the generator objective includes a matching discriminator loss $L_{\mathrm{match}}$. Together with the standard adversarial loss $L_{\mathrm{StyleGAN}3}\left(G,D\right)$, it forms the overall objective $L(G,D,D_{\mathrm{match}})$, as shown in Equation (6).

(5) $$\lambda_{\mathrm{match}}=\lambda_{\mathrm{mask}}\times \min\;\left(\frac{{\mathrm{cur}}_{\mathrm{kimg}}}{{\mathrm{warm}}_{\mathrm{kimg}}},\;1.0\right)$$

(6) $$L\left(G,D,D_{\mathrm{match}}\right)=L_{\mathrm{StyleGAN}3}\left(G,D\right)+\lambda_{\mathrm{match}}L_{\mathrm{match}}\left(G,D_{\mathrm{match}}\right)$$

where, as shown in Equation (5), the mask weight $\lambda_{\mathrm{match}}$ is scheduled to increase smoothly to its maximum and then remain constant, which prevents an overly substantial mask penalty at the beginning of training from impeding convergence. ${\mathrm{cur}}_{\mathrm{kimg}}$ denotes the current training progress (measured in kimg). $\lambda_{\mathrm{mask}}=0.1$, ${\mathrm{warm}}_{\mathrm{kimg}}=600$.

### 3.2. Defect Image Generation with Background Conditioning

For the same object, defect and background images are approximately drawn from a common underlying distribution, with only sparse deviations confined to the local regions indicated by the defect mask. In real-world scenarios, variations in surface treatments and optical conditions often invalidate the assumption that background and defect images share the same distribution, thereby inducing distributional shifts between them. Nevertheless, ImbDef-GAN remains effective in generating high-quality defects under varying conditions. By conditions defect generation on the background image and applies mask consistency and boundary smoothing constraints to generate defects in a controllable manner, alleviating sample scarcity while preserving background fidelity and natural transitions.

**Figure 4:**
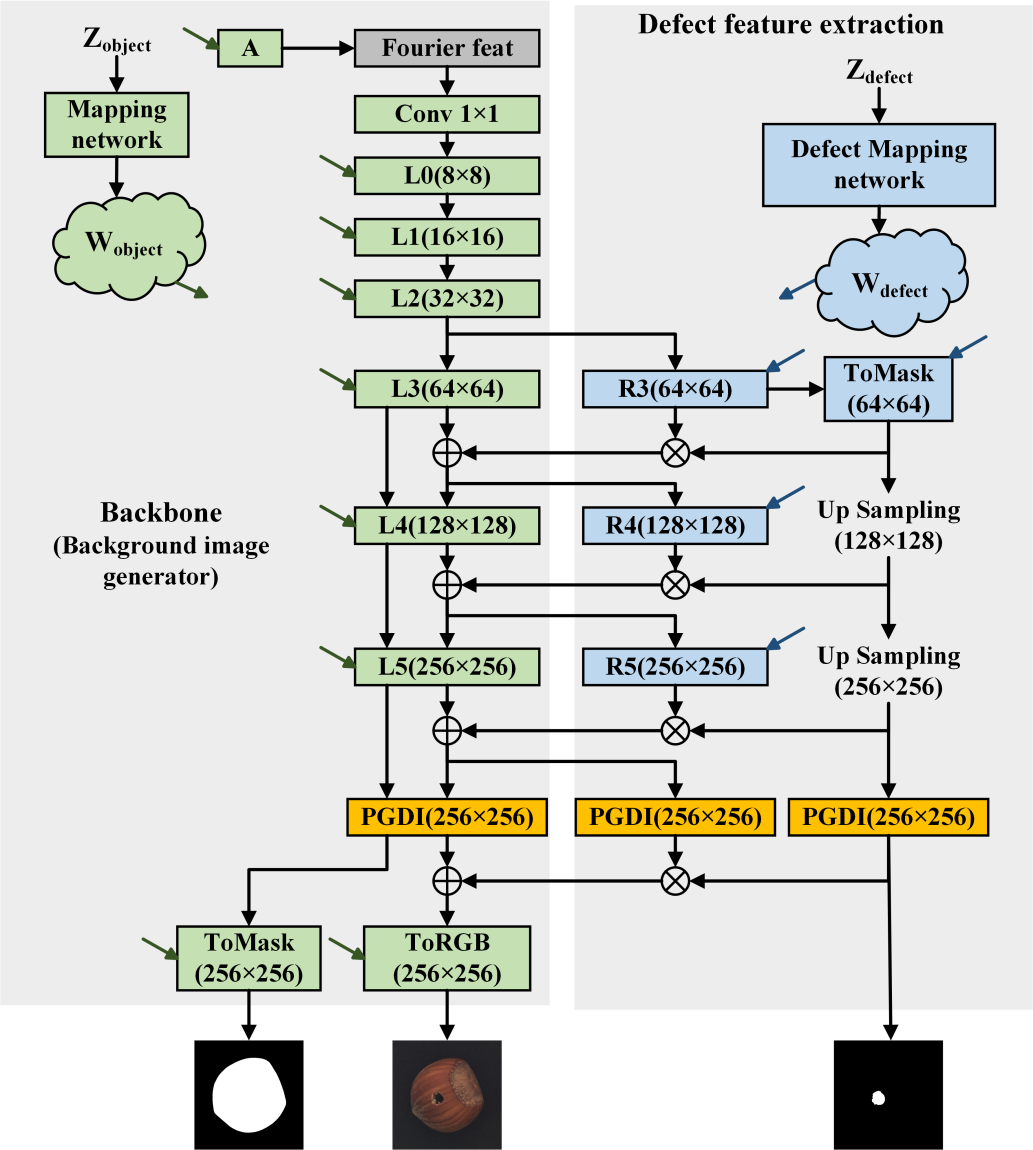
Architecture of the defect image generator.Green arrows indicate the $W_{\mathrm{object}}$ for background features, while blue arrows indicate the $W_{\mathrm{defect}}$ for defect features.

Therefore, fusing defect regions into background images offers an effective way to mitigate the scarcity of defect samples. To this end, ImbDef-GAN adopts a generator pretrained on abundant background images as the backbone and adds a residual branch for defect feature extraction. This branch comprises a defect mapping network and residual blocks for defect features. Features are first extracted at low resolutions and then passed through a ToMask module with progressive upsampling to produce the defect mask. The extracted defect features are subsequently blended with the background features using a smoothing coefficient, yielding the generated defect image. The detailed architecture is illustrated in Figure 4. During training, the backbone parameters are frozen so that the limited defective samples are devoted to learning defect-specific representations, thereby compensating for data scarcity.

Given that defect masks occupy a small portion of the image, existing matching discriminators tend to attenuate mask cues in later training, leading to spatial misalignment between generated defects and their corresponding masks. To mitigate this issue, a mask-aware matching discriminator is designed to continuously propagate mask information to deep feature layers, thereby strengthening image and mask alignment and improving the accuracy of defect localization. Meanwhile, Edge Structure Loss (ESL) encourages diverse boundary morphology to suppress boundary artifacts and structural monotony and to enhance overall realism; additionally, Region Consistency Loss (RCL) constrains the defect mask to the valid region defined by the background mask, preventing generation outside the object and avoiding invalid placement.

#### 3.2.1. Defect Feature Extraction

Defect feature extraction comprises two components: defect feature residual blocks and a defect mapping network. Within this module, the defect mapping network controls the morphological variation of defects. Compared with relying solely on object feature mappings in the backbone, the defect mapping network strengthens learning in defect regions, enabling more diverse defects, finer control of defect morphology and spatial distribution, and more natural generation of defect regions.

The defect feature residual block shares the architecture of the backbone’s generation blocks, placing defect and background features in a shared feature space and facilitating seamless fusion. A smoothing coefficient, denoted α, weights the fusion of defect and background features, producing more natural defect boundaries in the generated images. By freezing the backbone and training only the defect extraction branch on a small set of defect images, the model efficiently learns defect-specific representations, mitigating the challenge of defect image generation under few-sample conditions.

Let the generation block be denoted by *L* and the residual block by *R*. The first defect feature residual block R3 branches from the backbone-generation block at the 32 × 32 resolution. Each generation block output features $F_{\mathrm{object}}$, whereas each residual block output features $F_{\mathrm{defect}}$. Under this assumption, the backbone obtains background features $F_{\mathrm{object}}^{32}\in R^{N\times 32\times 32}$ before attaching the defect feature residual branch. Passing through the first defect feature residual block yields $F_{\mathrm{defect}}^{64}=R\;\left(F_{\mathrm{object}}^{32}\right)\in R^{N\times 64\times 64}$, whereas the corresponding generation block produces $F_{\mathrm{object}}^{64}=L\;\left(F_{\mathrm{object}}^{32}\right)\in R^{N\times 64\times 64}$; *N* denotes the number of channels. The ToMask module generates the defect region mask. Its architecture mirrors that of the backbone ToRGB module, differing only in the number of output channels. The ToRGB module outputs three channels, whereas ToMask outputs a single channel. The defect region mask, denoted $M=\mathrm{ToMask}\;\left(F_{\mathrm{defect}}^{64}\right)\in R^{1\times 64\times 64}$, delineates defect regions and guides subsequent processing of defect features.

After the mask is generated, Equation (7) binarizes the mask *M*, assigning 1 to defect pixels and 0 to background pixels. Equation (8) then computes, for each defect pixel, its distance to the defect boundary. To smooth the defect boundary, a smoothing coefficient α(i,j) is computed for pixels within the defect region using Equation (9). This coefficient is then used to weight the fusion of defect and background features: pixels farther from the boundary receive larger α(i,j) values, whereas pixels closer to the boundary receive smaller values. Consequently, the transition at the defect boundary is smoothed, yielding more natural edges in the generated images.

(7) $$M\left(i,j\right)=\left\{\begin{array}{cc} 1 & M(i,j)\geq 0\\ 0 & M(i,j)<0 \end{array}\right.$$

(8) $$d\left(i,j\right)=\min_{(u,v)\in \Omega }\left(|i-u|+|j-v|\right)$$

(9) $$\alpha \left(i,j\right)=1-\exp\;\left(-\frac{d{\left(i,j\right)}^{2}}{2\sigma^{2}}\right)$$

where Ω={(u,v)∣mask(u,v)=0}. M(i,j) denotes a specific location on the mask. For each defect pixel, let d(i,j) denote its distance to the nearest boundary of the defect region. The parameter σ controls the edge transition bandwidth, defined as the range over which the smoothing coefficient increases toward 1. Increasing σ widens and slows the transition, whereas decreasing σ narrows and accelerates it; in this work, we set σ to 2.0. After computing the per-pixel weights inside the defect region, the smoothed coefficient map $F_{\mathrm{object}}^{64}$ is updated according to Equation (10).

(10) $$F_{\mathrm{object}}^{64}\left(i,j\right)=\left\{\begin{array}{cc} \left(1-\alpha \left(i,j\right)\right)\cdot F_{\mathrm{object}}^{64}\left(i,j\right)+\alpha \left(i,j\right)\cdot F_{\mathrm{defect}}^{64}\left(i,j\right) & M(i,j)\geq \gamma \\ F_{\mathrm{object}}^{64}\left(i,j\right) & M(i,j)<\gamma \end{array}\right.$$

where the threshold γ is set to 0, and (i,j) denotes a specific location on the feature map or the defect mask. With this design, features near the defect boundary are blended with background features in a weighted manner, yielding smoother boundary transitions. At resolutions of 64 × 64 and 128 × 128, the mask *M* is upsampled from the previous stage to the corresponding resolution; the defect feature residual blocks at these scales follow the same procedure as at 32 × 32 when processing the target feature map within the defect region.

#### 3.2.2. Mask-Aware Matching Discriminator

In defect imagery, the anomalous region typically occupies a small fraction of pixels, so reusing a background-oriented matching discriminator often attenuates mask cues in the final layers and limits precise correspondence between the image and the mask. This motivates the introduction of a mask-aware matching discriminator that forms a four-channel input by concatenating the RGB image with its mask and progressively injects a downsampled mask at each stage.

**Figure 5:**
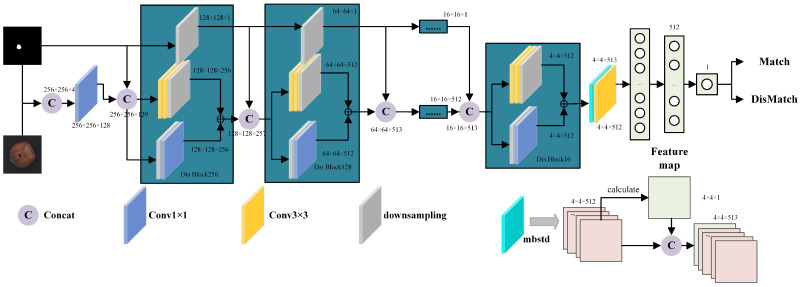
Architecture of the mask-aware matching discriminator.

As shown in Figure 5, the discriminator concatenates the three-channel defect image with the one-channel mask to form a four-channel input. The input then passes through a multi-stage convolutional downsampling path that progressively reduces spatial resolution and extracts multi-scale features for evaluating consistency between the image and the mask. At each stage, the mask is downsampled to the current resolution and concatenated with the feature map, injecting spatial cues that guide representation learning across the network. Residual connections strengthen cross-layer information flow and improve training stability and convergence. Together, these designs yield finer and more accurate alignment between generated images and masks across multiple spatial scales.

The minibatch standard deviation (mbstd) module at the discriminator tail enhances the discriminator’s sensitivity to sample diversity. It first partitions the input features according to a specified group size and number of channels, then computes the standard deviation across channel and spatial dimensions within each group, yielding a feature map that captures intra-minibatch statistical fluctuations. This map is concatenated with the original features along the channel dimension and passed to subsequent discriminator layers, which improves sensitivity to variation among generated samples and helps mitigate mode collapse in the generator.

The mask-aware matching discriminator adopts Wasserstein adversarial loss [34] and is optimized with regularization. Working in concert with the StyleGAN3 discriminator during defect image generation, it yields more realistic and diverse images and achieves higher localization accuracy for defect regions.

#### 3.2.3. Edge Structure Loss

The generated defect image is jointly determined by object and defect features. In practice, if defect features exert only a limited influence on the final defect morphology, different defect feature mappings may produce negligible variations, thereby constraining the diversity of generated defects. To prevent weak variations and encourage distinct morphologies under different defect conditions, Edge Structure Loss (ESL) is introduced, inspired by mode seeking [35]. ESL promotes diversity in the space of defect shape and geometry rather than raw intensity, aligning latent perturbations with meaningful structural changes.

ESL employs the Laplacian edge operator to extract edge structure from defect masks, aiming to elevate the discrepancy measure from simple pixel-level differences to structural edge-level differences, thereby guiding the generator to focus more on structural variations in defect morphology. As shown in Equation (11), the edge operator effectively captures boundary variation in masks and further increases the structural diversity of generated defect masks. Although the Laplacian operator is a conventional image processing tool, it remains stable and efficient on defect masks, whose structures are well defined with sharp boundaries. The detailed formulation of ESL is given in Equation (12).

(11) $$E\left(M\right)=\mathrm{Laplacian}\left(M\right)=M*K,\;K=\left[\begin{array}{ccc} 0 & 1 & 0\\ 1 & -4 & 1\\ 0 & 1 & 0 \end{array}\right]$$

(12) $$L_{\mathrm{ES}}=\frac{∥W_{1}-W_{2}{∥}_{1}}{∥E\left(M_{1}\right)-E\left(M_{2}\right){∥}_{1}+\varepsilon }$$

$W_{1}$ and $W_{2}$ are the generated defect images obtained from random defect codes $Z_{1}$ and $Z_{2}$ via the defect mapping network, while $M_{1}$ and $M_{2}$ denote their corresponding defect masks. A small constant ε is introduced to avoid division by zero, $\varepsilon =10^{-6}$.

#### 3.2.4. Region Consistency Loss

In the background image generation, background masks are generated. To ensure that generated defect regions remain confined within valid areas of the background image, Region Consistency Loss (RCL) enforces this constraint and injects an explicit spatial validity prior into training, which reduces artifacts outside the background area and improves localization accuracy. RCL penalizes pixels of the defect mask that extend beyond the valid region of the background mask, thereby constraining defect features to appear only within valid background regions and ensuring that defect locations remain consistently within the effective area of the background image. After obtaining the background mask, RCL follows Equation (15).

(13) $$M_{\mathrm{bg}}\left(i,j\right)=\left\{\begin{array}{cc} +1 & M_{\mathrm{bg}}\left(i,j\right)\geq 0\\ -1 & M_{\mathrm{bg}}\left(i,j\right)<0 \end{array}\right.$$

(14) $$I=\frac{1-M_{\mathrm{bg}}}{2}$$

(15) $$L_{\mathrm{RC}}=\frac{\sum_{(i,j)\in \Omega }I\left(i,j\right)\;{\left[\text{max}\;\left(M_{\mathrm{defect}}\left(i,j\right)-\tau ,0\right)\right]}^{2}}{\sum_{(i,j)\in \Omega }I\left(i,j\right)}$$

where Ω denotes the set of mask pixels; $M_{\mathrm{bg}}\left(i,j\right)$ is the pixel value at location (i,j) in the background mask. It is preprocessed by Equation (13) to +1 (valid region) or −1 (invalid region). $M_{\mathrm{defect}}\left(i,j\right)$ denotes the pixel value at the exact location in the generated defect mask. A squared penalty is applied if and only if a pixel satisfies $M_{\mathrm{bg}}\left(i,j\right)=-1$ (invalid background region) and $M_{\mathrm{defect}}\left(i,j\right)>\tau$, where τ is the tolerance threshold. By summing and normalizing the squared errors over all out-of-bound pixels, the model enforces that defects are strictly confined to the valid foreground regions of the background mask, while maintaining smooth and stable training.

Finally, the ESL and RCL are integrated with the other GAN related losses to form the overall defect image-generation objective, as defined in Equation (16). Together, these losses guide the generator and discriminator to improve defect localization during training, thereby enhancing the realism and diversity of defects with respect to the background.

(16) $$L\left(G,D,D_{\mathrm{match}}\right)=L_{\mathrm{StyleGAN}3}\left(G,D\right)+L_{\mathrm{match}}\left(G,D_{\mathrm{match}}\right)+\lambda_{\mathrm{ES}}L_{\mathrm{ES}}\left(G\right)+L_{\mathrm{RC}}$$

where $\lambda_{ES}=0.1$; $L_{\mathrm{StyleGAN}3}\left(G,D\right)$ denotes the standard StyleGAN3 loss, which optimizes the generator *G* and the discriminator *D*. $L_{\mathrm{match}}\left(G,D_{\mathrm{match}}\right)$ is used to optimize the generator *G* and the mask-aware matching discriminator $D_{\mathrm{match}}$, and its computation follows the same formulation as $L_{\mathrm{StyleGAN}3}\left(G,D\right)$.

## 4. Experiments and Results

To validate the effectiveness of the proposed method, we conduct evaluations on the MVTec AD dataset [36] from two perspectives: background image generation and defect image generation.

### 4.1. Experiments Setup

#### 4.1.1. Dataset: MVTec AD

The MVTec Anomaly Detection (MVTec AD) dataset [36] comprises 10 object categories and five texture categories, each with up to 8 defect types, and provides pixel-level defect masks. Although originally designed for defect localization, most categories in MVTec AD contain 200–400 background samples but only 10–25 images per defect type, making it well suited to defect image generation under severe class imbalance.

ImbDef-GAN is evaluated on the hazelnut, bottle, and metal_nut categories of the MVTec AD dataset, as summarized in Table 2, to assess its ability to generate defects across diverse object types and its cross-category adaptability. Hazelnut, bottle, and metal_nut represent natural materials, transparent media, and metallic parts, respectively. Together, these categories span the random textures and irregular boundaries of natural objects, as well as the sharp edges and high frequency perturbations typical of industrial components. Consequently, their complementary structural and morphological properties enable a comprehensive evaluation of the model’s generative quality under class imbalance.

#### 4.1.2. Implementation Details

ImbDef-GAN was implemented in PyTorch 1.13 with Python 3.10, CUDA 11.7. All experiments are conducted in a Windows environment using an NVIDIA GeForce RTX 4090 GPU for both training and testing, and a complete training run for one object category, covering both background and defect image training, takes approximately 2.8 days. The training data are drawn from the MVTec AD dataset [36], with images resized to a resolution of 256 × 256. The Adam optimizer is employed with an initial learning rate of 0.0025 and a batch size of 32. The key hyperparameters for each module are described in detail in the corresponding sections. In addition, weight normalization and learning rate scheduling are applied during training to enhance stability and accelerate convergence.

#### 4.1.3. Evaluation Metrics

To evaluate the diversity and realism of the generated images, three metrics are employed: Fréchet Inception Distance (FID) [37], Kernel Inception Distance (KID) [38], and Learned Perceptual Image Patch Similarity (LPIPS) [39].

For evaluating background image generation, FID serves as the primary metric. A lower FID score indicates greater realism of the generated background images. The formulation of FID is given in Equation (17).

(17) $$\mathrm{FID}\left(x,y\right)=∥{\mu_{x}-\mu_{y}∥}^{2}+\mathrm{Tr}\;\left(\Sigma_{x}+\Sigma_{y}-2{\left(\Sigma_{x}\Sigma_{y}\right)}^{\frac{1}{2}}\right)$$

where $\mu_{x}$ denotes the mean feature vector extracted from real images *x* using the Inception-v3 network [40], and $\mu_{y}$ denotes the corresponding mean feature vector from generated images *y*. $\Sigma_{x}$ represents the covariance matrix of features extracted from *x*, while $\Sigma_{y}$ represents the covariance matrix of features extracted from *y*.

Defect image generation is primarily evaluated using KID and LPIPS. Because real defect samples are scarce, FID yields biased estimates in few-sample settings, leading to unstable evaluations. By contrast, KID uses an unbiased maximum mean discrepancy (MMD) estimator, offering greater robustness and stability and making it better suited to assessing image quality and diversity in few-shot scenarios. Accordingly, KID is the primary quality metric for reliably reflecting distributional differences between generated and real defect images, with LPIPS providing a complementary perceptual measure.

(18) $${\mathrm{MMD}}^{2}\left(X,Y\right)=\frac{1}{m(m-1)}\sum_{i\neq j}k\left(x_{i},x_{j}\right)+\frac{1}{n(n-1)}\sum_{i\neq j}k\left(y_{i},y_{j}\right)-\frac{2}{mn}\sum_{i=1}^{m}\sum_{j=1}^{n}k\left(x_{i},y_{j}\right)$$

(19) $$k\left(x,y\right)={\left(\frac{1}{d}x^{T}y+1\right)}^{3}$$

where *m* and *n* denote the number of generated and real samples, respectively, and *d* is the dimensionality of the feature vectors. Both *x* and *y* are feature vectors extracted from the Inception-v3 network.

For each defect category, 5000 samples are generated and compared with the corresponding authentic defect images in the dataset to compute the KID score. A lower KID score indicates that the generated images are closer to real ones in terms of realism and diversity, reflecting higher overall quality.

LPIPS is a metric for measuring perceptual similarity between images and is particularly suited for evaluating the diversity of generated samples. Suppose a given defect category in the dataset contains *N* images. For 1000 generated images, the evaluation first computes their LPIPS similarity to the closest real image in the dataset and then assigns each generated image to one of the *N* clusters accordingly. Next, the evaluation obtains the cluster mean LPIPS by averaging over all image pairs within each cluster, and then averages these cluster means to yield the overall mean. A higher cluster-level LPIPS score indicates greater diversity among the generated images. Combining KID and LPIPS provides a more comprehensive evaluation of both the realism and diversity of generated defect images.

### 4.2. Background Image Generation Experiments

Our goal is to generate defect images that exhibit both realism and diversity. Since our approach generates defects on top of background images, the quality of background image generation directly determines the quality of defect image generation. To this end, we compare StyleGAN2 [16], StyleGAN3 [17], a lightweight variant (LStyleGAN3), and our proposed LStyleGAN3 with the PGDI module, in order to evaluate background image-generation quality and thereby ensure reliable defect image generation.

As shown in Table 3, for the hazelnut category, we obtain the lowest FID of 13.59 across five independent runs, with the highest value reaching 14.95, indicating more realistic background image generation. The lightweight variant LStyleGAN3 attains a best FID of 17.05, outperforming StyleGAN3 and StyleGAN2, which record 19.23 and 20.41, respectively, suggesting that the lightweight redesign preserves generation quality while better meeting the requirements of background image generation.

**Figure 6:**
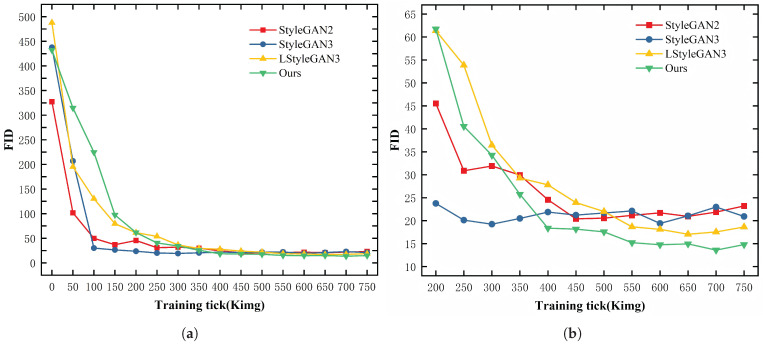
(**a**) FID trajectory of background image generation over training. (**b**) Zoomed view from 200 to 750 ticks highlighting the convergence phase.

Figure 6a shows the FID trajectory during training for the compared methods. Because FID stabilizes below 65 after 200 ticks, Figure 6b focuses on the 200–750 tick interval to highlight differences among the models. All four methods drop below an FID of 50 after 300 ticks. StyleGAN2 and StyleGAN3 exhibit a rebound in FID in mid-to-late training, whereas LStyleGAN3 continues to decrease, reaching 17.05 at 650 ticks. Our method exhibits a smoother FID descent with fewer fluctuations. It continues to decrease, attaining a global minimum of 13.59 at 700 ticks and remaining as low as 14.79 at 750 ticks. With progress-aware scheduling and pixel-wise gating in PGDI, the model avoids early excessive injection of high frequency detail that would otherwise destabilize training. Once global structure and low frequency contours stabilize, PGDI enhances salient details, yielding more effective late stage reductions and a lower FID. The resulting stability and detail fidelity provide a robust foundation for subsequent alignment between images and masks and for high quality defect image generation.

### 4.3. Defect Image Generation Experiments

#### 4.3.1. Multi-Class Defect Image Generation on a Shared Background

**Figure 7:**
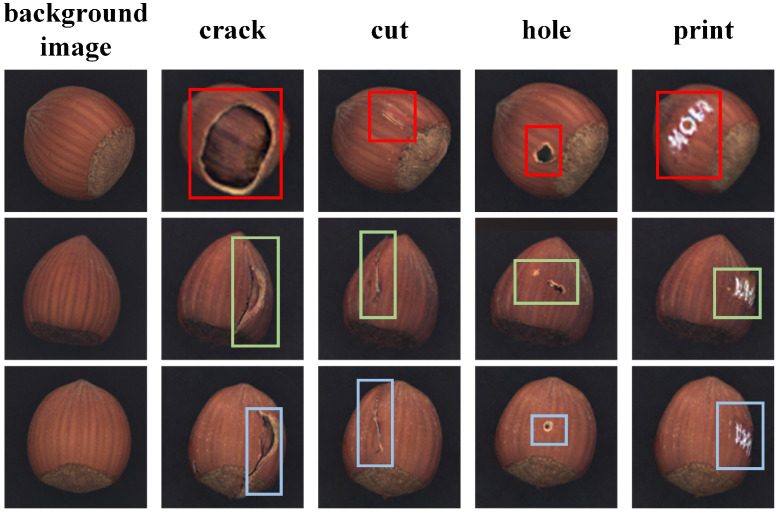
Generation of multiple defect types on the same background. Different-colored boxes denote defects from different background images.

Our defect image generation relies on background images, onto which we attach distinct defect feature regions. In this manner, a single background can yield multiple types of defect images. Figure 7 presents category conditioned generation. A background is first generated, after which category specific defect features are injected to obtain exemplars for multiple defect types. The qualitative results indicate that the model produces diverse and realistic defects under limited defect supervision, and that defect appearance adapts to the surrounding background with coherent context. This approach alleviates scarcity across categories, especially under class imbalance, by enabling the conditional generation of diverse defect images on a shared background.

#### 4.3.2. Comparative Experiments on Defect Image Generation

For defect image generation, we compare ImbDef-GAN against seven methods. Crop&Paste [11] is a classic data-augmentation method. StyleGAN2 [16] and StyleGAN3 [17] are canonical generative models. Compared with conventional GANs, StyleGAN2 offers higher image quality and training stability, better captures fine grained textures, and supports controllable style manipulation. StyleGAN3 refines the generation process to suppress aliasing and periodic artifacts, yielding smoother and more consistent textures at high resolution and improving the realism of fine details. Defect-GAN [21], which follows a damage–recovery pipeline with a layer-wise generator and does not jointly generate defect masks, generates highly realistic defects across backgrounds with diverse textures and appearances. DFMGAN [18] pretrains on normal images, fine-tunes on defective images, and inserts defects by pixel-level pasting or addition; with transfer learning it produces realistic defects when samples are scarce. AnomalyDiffusion [19] is a diffusion-based method that decouples defect appearance from mask distribution and generates defects at specified mask locations on normal images, enhancing diversity. He et al. [24] proposed a dual stream StyleGAN2-ADA pipeline that uses indirect decoupling and mask guided attention to synthesize diverse, defect centered images, thereby improving anomaly and defect detection on MVTec AD. Table 4 compares methods using KID and LPIPS for defect image generation. Our method achieves the best KID and LPIPS scores (lower KID, higher LPIPS), confirming its advantage for defect image generation under class imbalanced conditions.

By comparison, defects generated by Crop&Paste closely track the dataset distribution, yielding KID ≈ 0; therefore, the KID entries are omitted in Table 4. StyleGAN2 and StyleGAN3 tend to overfit, which limits the diversity of generated defects; their LPIPS values are typically below 0.1. Although Defect-GAN achieves KID ≈ 30, the generated defect cues are weak. Under scarce defect samples, it still struggles to produce high quality defects, with LPIPS ≈ 0.2. DFMGAN and AnomalyDiffusion face a trade off between realism and diversity: DFMGAN and the few-shot method by He et al. using StyleGAN2-ADA prioritize realism, whereas AnomalyDiffusion yields greater diversity. Our approach preserves diversity while further improving realism in the defect regions. Compared with DFMGAN, our method reduces KID by 6.13 and 10.24 on the hole and print categories, respectively. Despite limited defect samples, the proposed method demonstrates strong defect-generation capability and achieves superior performance in both realism and diversity.

**Figure 8:**
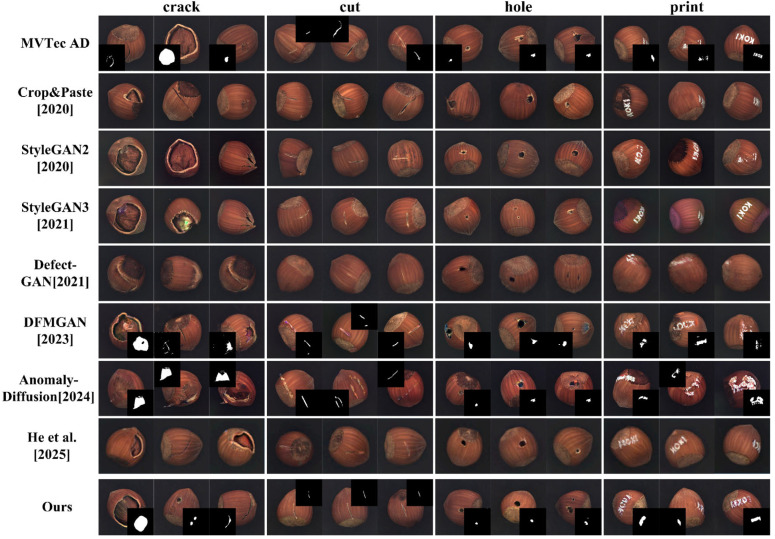
Comparison of defect images generated on the hazelnut. Crop&Paste [2020] [11], StyleGAN2 [2020] [16], StyleGAN3 [2021] [17], Defect-GAN [2021] [21], DFMGAN [2023] [18], AnomalyDiffusion [2024] [19], He et al. [2025] [24].

Figure 8 provides a qualitative comparison on the hazelnut category. The top row presents real defect images and masks from MVTec AD as a reference. The subsequent rows display results from different methods in a fixed order, and each column corresponds to a distinct defect subcategory.

Defects generated by Crop&Paste more closely match the real data distribution. Although StyleGAN2 and StyleGAN3 perform well on general image generation, they are less effective with scarce defect data. On hazelnut, the generated defects show overfitting, limited diversity, and inflated structural artifacts. In addition, StyleGAN3 exhibits pronounced color shifts, reducing realism. With limited defect samples, Defect-GAN struggles to capture salient characteristics of defect regions, yielding defects that are insufficiently prominent and overly small, which falls short of practical requirements. DFMGAN offers advantages over these methods, but its defect boundaries remain unnatural. Misalignment between defect masks and generated regions further degrades realism and limits the quality of the generated regions. AnomalyDiffusion achieves greater diversity but lacks realism in defect regions. Generated defects sometimes spill beyond valid areas. The feature disentanglement method based on StyleGAN2-ADA proposed by He et al. generates defect images that closely approximate the appearance of real defects. Our method outperforms existing approaches across multiple categories, mitigating unnatural boundary texture and morphology, misalignment between images and masks, and placement outside valid regions. The generated results align more closely with real defect images in both overall quality and fine grained detail.

#### 4.3.3. Comparative Experiments for 5-Shot and 1-Shot Defect Image Generation

**Figure 9:**
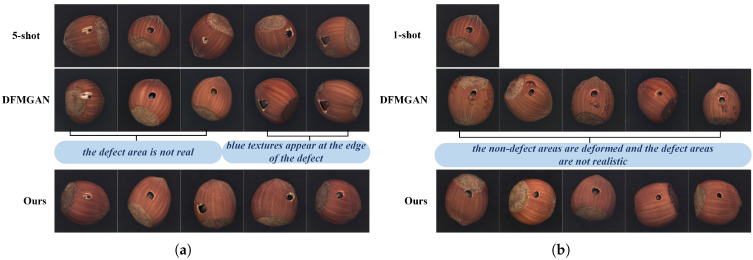
(**a**) Comparison of defect images generated using 5-shot. (**b**) Comparison of defect images generated using 1-shot.

In the MVTec AD hazelnut category [36], the hole defect class contains 18 defect images in total. To compare with DFMGAN [18] with limited data, 5-shot and 1-shot settings are evaluated by selecting 5 and 1 defect images, respectively, from the 18 available. With five training images, as shown in Figure 9a, DFMGAN exhibits pronounced color shifts in generated defect regions. Bluish artifacts often appear around defects, indicating limited fidelity to real morphology and texture. With a single training image, as shown in Figure 9b, these issues intensify: defect regions lack realism, and the background shows deformation, artifacts, and blur, collectively reducing overall fidelity. By comparison, our method performs better under data scarcity, producing defect regions that more closely match real morphology and texture and offering higher visual realism and discriminability.

#### 4.3.4. Comparative Experiments on Defect Image Generation in Other Scenarios

**Figure 10:**
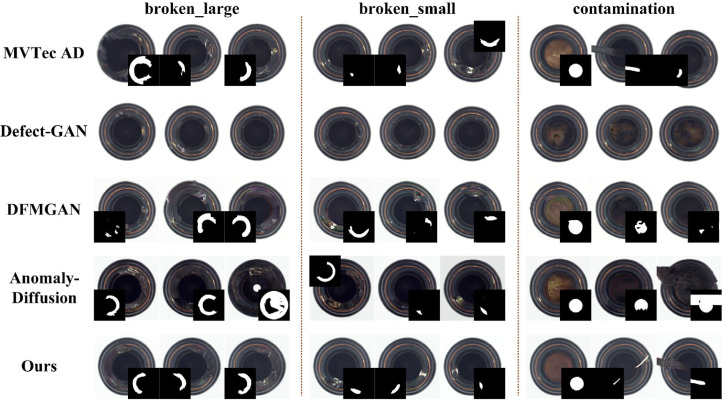
Comparison of defect images generated on the bottle.

**Figure 11:**
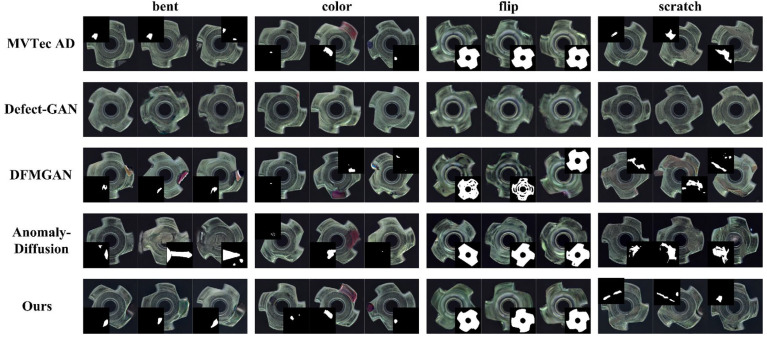
Comparison of defect images generated on the metal_nut.

To assess generalization across scenarios, we conduct comparative experiments on the MVTec AD bottle and metal_nut categories [36]. Results are summarized in Table 5 and Figure 10 and in Table 6 and Figure 11.

Although Defect-GAN [21] performs well in realism, the generated defects lack salient characteristics in both categories. DFMGAN [18] exhibits unnatural boundary transitions in both categories. Defect edges often show color discontinuities and halo artifacts, and the generated defects are not precisely aligned with their masks. AnomalyDiffusion [19] achieves higher appearance diversity but sometimes places defects implausibly, and local realism in defect regions is limited. Our method consistently generates defect regions with intact structure and natural boundary transitions. The generated masks align accurately with the defects, yielding higher overall realism. Consequently, the proposed approach shows good generalization and practical applicability within the evaluated settings, making it a promising option for defect image generation across the examined scenarios.

#### 4.3.5. Ablation Study

We conduct ablation studies on the hazelnut/hole category, comparing five model variants: (1) ResBlock32—extract defect features at 32 × 32 resolution; (2) ResBlock128—extract defect features at 128 × 128 resolution; (3) NoMAMatch—replace the mask-aware matching discriminator with a StyleGAN3 structured matching discriminator; (4) NoMS—remove the Edge Structure Loss; and (5) NoRC—remove the Region Consistency Loss.

As shown in Table 7, extracting defect features at 64 × 64 achieves a better trade-off between detail fidelity and overfitting. It strengthens the representation of defect regions while maintaining stable training and higher perceptual diversity. Replacing the discriminator reduces both KID and LPIPS. Propagating mask information through all discriminator layers aligns spatial semantics and improves correspondence between images and masks, enabling the generator to learn more accurate defect morphology and fine details. Edge Structure Loss primarily governs defect diversity, increasing the diversity metric by 0.083 and encouraging richer and finer structural variation in defect regions. Region Consistency Loss prevents defects from spilling into invalid background areas, which would otherwise degrade both realism and diversity.

### 4.4. Comparative Experiments on Defect Detection

YOLO [5] is a representative single stage, end-to-end detector noted for high throughput and low latency. For defect detection, YOLOv11 [25] enhances multi-scale feature representation and the detection head, markedly improving detection rate and localization accuracy for small scale, low contrast, fine grained defects. Accordingly, we adopt YOLOv11 to assess the gains from the proposed defect image-generation method and evaluate it under different generation strategies. To ensure objectivity and reliability, the test set satisfies three criteria: (i) it is disjoint from all data used for image generation; (ii) it contains only real images, with no generated samples; and (iii) its defect category distribution matches the real world distribution.

The dataset is split into training and test sets, with one-third for training and two-thirds for testing. The training set is used to generate 200 images per defect category (800 in total) to augment detector training. The categories and counts are summarized in Table 8.

Differences in defect-detection performance across datasets are quantified using mean Average Precision (mAP). mAP is the mean of per-class average precision (AP), where each AP is the area under the precision–recall (PR) curve; it summarizes per-class detection performance across categories. Higher mAP indicates better localization and recognition of defects. In our setting, higher mAP suggests that generated defect images preserve spatial placement, show natural boundary transitions, and encode discriminative features, enabling more effective recognition and localization. Accordingly, mean Average Precision (mAP) serves as the primary metric to quantify gains from defect image generation. To assess overall detection performance across categories, mAP@0.5, defined as the mean of average precision computed per class at an IoU threshold of 0.5, is reported.

**Figure 12:**
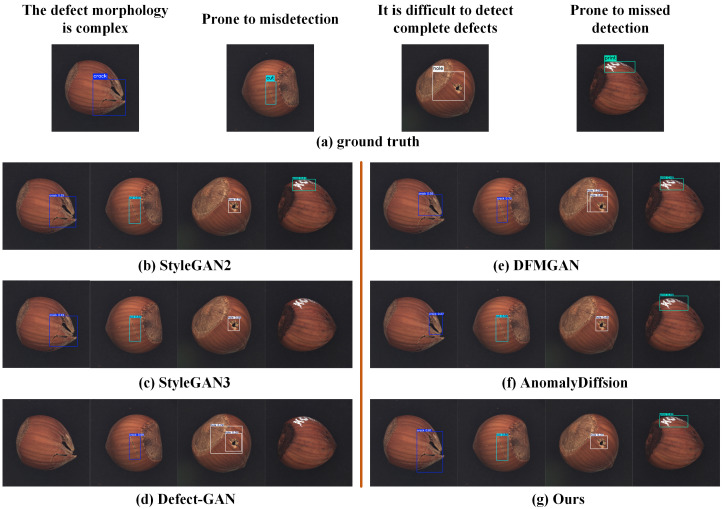
Visualization of selected detection results.

Table 9 reports mAP@0.5 for detectors trained with defect images generated by different methods. ImbDef-GAN attains 83.6%, the best result, surpassing AnomalyDiffusion at 78.2% by 5.4 points and DFMGAN at 76.1% by 7.5 points. To assess adaptability in complex real-world scenarios, Figure 12 presents four challenging cases: (i) complex defect morphology; (ii) defects with appearance similar to other classes; (iii) regions that are difficult to detect completely; and (iv) weak cues prone to omission. In these settings, our approach accurately localizes defects and correctly correctly classifies their categories. Even under high inter-class similarity, it maintains strong detection performance. The method generates defect images with high realism and diversity, which enhances detection robustness and reduces both false positives and missed detections.

## 5. Conclusions

This work addresses three challenges in defect image generation: unnatural defect boundaries, imprecise alignment between generated images and their masks, and implausible defect placement. Abundant background images together with scarce defect images are leveraged to tackle these issues. We propose ImbDef-GAN, a generative framework that accounts for sample imbalance and integrates defect features into generated background images to generate defect images. First, a lightweight variant of the StyleGAN3 [17] generator is designed to jointly generate the background and its mask, and their consistency is enforced with a matching discriminator. A Progress-coupled Gated Detail Injection module enhances background detail while preserving training stability. Second, a residual branch extracts defect features and blends them with background features under a smoothing coefficient, yielding more natural and gradual defect boundaries. To improve alignment between images and masks, a mask-aware matching discriminator propagates mask information through multilayer feature extraction. We further introduce Edge Structure Loss to emphasize boundary morphology and Region Consistency Loss to suppress defects in invalid background areas. Experiments on the MVTec AD dataset [36] indicate consistent gains in realism and diversity; nevertheless, performance remains sensitive to the quality of the jointly generated background mask, particularly in scenes with ambiguous object boundaries or occlusion. Training a YOLOv11 [25] detector on the generated data yields measurable accuracy gains and reduces both false positives and false negatives. However, in real-world scenarios, the assumption of distributional consistency between defects and backgrounds does not always hold, so localization of tiny defects and fine textures may still be limited under extreme conditions.

## Figures and Tables

**Table 1:** Comparison of data-augmentation methods for defect images.

Methods	Advantages	Limitations
Transformation-based augmentation (scaling, rotation, translation, flipping, etc.)	Simple and efficient; computationally lightweight	Cannot faithfully model realistic and complex defect structures
Traditional defect image generation (CutPaste, Crop&Paste, etc.)	Simple and highly controllable; capable of producing localized structural perturbations	Limited realism; inadequate coverage of complex morphologies; constrained by source images
Deep learning–based defect image generation (DFMGAN, AnomalyDiffusion, etc.)	Generalize across defect types and learn realistic; complex defect structures	Training is complex and may suffer from mode collapse or instability

**Table 2:** Defect categories of selected subsets from the MVTec AD dataset.

Hazelnut		Bottle		Metal_Nut	
Defect Category	Count	Defect Category	Count	Defect Category	Count
crack	18	broken_large	20	bent	25
cut	17	broken_small	22	color	22
hole	18	contamination	21	flip	23
print	17	–	–	scratch	23

**Table 3:** Comparison of evaluation metrics for background image generation of the hazelnut (↓ lower is better).

Methods	FID ↓
StyleGAN2	20.41
StyleGAN3	19.23
LStyleGAN3	17.05
Ours	14.27 ± 0.68

**Table 4:** Comparison of KID and LPIPS evaluation results for defect image generation on the hazelnut, where KID $\times \;10^{3}@5k$ and LPIPS@1k are reported (↓ lower is better; ↑ higher is better).

Hazelnut	Crack		Cut		Hole		Print	
Methods	KID↓	LPIPS↑	KID↓	LPIPS↑	KID↓	LPIPS↑	KID↓	LPIPS↑
Crop&Paste [11]	–	0.1894	–	0.2045	–	0.2108	–	0.2185
StyleGAN2 [16]	22.51	0.0548	18.58	0.0734	30.81	0.0734	19.61	0.0842
StyleGAN3 [17]	36.93	0.0891	60.60	0.0712	103.94	0.1370	135.15	0.0971
Defect-GAN [21]	30.98	0.1905	32.69	0.1734	36.30	0.2007	33.35	0.2007
DFMGAN [18]	19.73	0.2600	16.88	0.2073	20.78	0.2391	27.25	0.2649
AnomalyDiffusion [19]	32.59	0.3111	21.19	0.2753	29.40	0.2846	31.01	0.3139
He et al. [24]	15.83	0.2759	14.44	0.2263	20.32	0.2521	19.34	0.2359
Ours	16.28	0.3261	12.59	0.2851	14.65	0.3065	17.01	0.3112

**Table 5:** Comparison of KID and LPIPS evaluation results for defect image generation on the bottle, where KID $\times \;10^{3}@5k$ and LPIPS@1k are reported (↓ lower is better; ↑ higher is better).

Bottle	Broken_Large		Broken_Small		Contamination	
Methods	KID↓	LPIPS↑	KID↓	LPIPS↑	KID↓	LPIPS↑
Defect-GAN [21]	77.09	0.0593	59.18	0.0797	126.45	0.0693
DFMGAN [18]	59.74	0.1162	76.38	0.0854	76.59	0.1661
AnomalyDiffusion [19]	82.26	0.1898	75.49	0.1646	73.86	0.1766
Ours	56.32	0.1652	55.25	0.1536	65.03	0.1884

**Table 6:** Comparison of KID and LPIPS evaluation results for defect image generation on the metal_nut, where KID $\times \;10^{3}@5k$ and LPIPS@1k are reported (↓ lower is better; ↑ higher is better).

Metal_Nut	Bent		Color		Flip		Scratch	
Methods	KID↓	LPIPS↑	KID↓	LPIPS↑	KID↓	LPIPS↑	KID↓	LPIPS↑
Defect-GAN [21]	55.94	0.3058	44.83	0.3138	148.86	0.2836	56.29	0.3063
DFMGAN [18]	34.14	0.3153	35.72	0.3326	67.66	0.2919	38.65	0.3315
AnomalyDiffusion [19]	46.28	0.2921	32.23	0.2644	74.51	0.3223	35.39	0.2927
Ours	29.22	0.3254	30.52	0.3529	74.32	0.3027	33.95	0.3453

**Table 7:** Comparative results of the ablation study (↓ lower is better; ↑ higher is better).

Methods	KID ↓	LPIPS ↑
(1) ResBlock32	23.75	0.2370
(2) ResBlock128	16.47	0.2621
(3) NoMAMatch	20.65	0.2642
(4) NoMS	15.60	0.2235
(5) NoRC	17.32	0.2751
(6) Ours (ResBlock64)	14.65	0.3065

**Table 8:** Data distribution across the dataset.

Defect Category	Training Set (Images)		Testing Set (Images)
	Original Count	Generated Count	Original Count
crack	6	200	12
cut	5	200	12
hole	6	200	12
print	5	200	12
Total	22	800	48

**Table 9:** Comparison of evaluation metrics (mAP@0.5) using generated data (↑ higher is better).

Methods	mAP@0.5/% ↑
StyleGAN2	65.5
StyleGAN3	50.9
Defect-GAN	65.2
DFMGAN	76.1
AnomalyDiffusion	78.2
Ours	83.6

## Data Availability

The original contributions presented in this study are included in the article. Further inquiries can be directed to the corresponding author.
